# Unilateral biportal endoscopic lumbar interbody fusion assisted by intraoperative O-arm total navigation for lumbar degenerative disease: A retrospective study

**DOI:** 10.3389/fsurg.2022.1026952

**Published:** 2022-09-23

**Authors:** Xinle Huang, Junfeng Gong, Huan Liu, Zegang Shi, Wenkai Wang, Shuai Chen, Xiaobing Shi, Changqing Li, Yu Tang, Yue Zhou

**Affiliations:** Department of Orthopedics, Xinqiao Hospital, Army Millitary Medical University, Chongqing, China

**Keywords:** intraoperative o-arm, total navigation, unilateral biportal endoscopic lumbar interbody fusion, minimally invasive spine, lumbar degenerative diseases

## Abstract

**Background:**

Recently, unilateral biportal endoscopic lumbar interbody fusion (BE-LIF) has been successfully applied for degenerative diseases of the lumbar spine, with good clinical results reported. However, the drawbacks include radiation exposure, limited field of view, and steep learning curves.

**Objective:**

This retrospective study aimed to compare the results between navigation and non-navigation groups and explore the benefits of BE-LIF assisted by intraoperative O-arm total navigation.

**Methods:**

A total of 44 patients were retrospectively analyzed from August 2020 to June 2021. Perioperative data were collected, including operative time, estimated intraoperative blood loss, postoperative drainage, postoperative hospital stay, radiation dose, and duration of radiation exposure. In addition, clinical outcomes were evaluated using postoperative data, such as the Oswestry Disability Index (ODI), visual analog scale (VAS), modified MacNab criteria, Postoperative complications and fusion rate.

**Results:**

The non-navigation and navigation groups included 23 and 21 patients, respectively. All the patients were followed up for at least 12 months. No significant differences were noted in the estimated intraoperative blood loss, postoperative drainage, postoperative hospital stay, fusion rate, or perioperative complications between the two groups. The radiation dose was significantly lower in the navigation group than in the non-navigation group. The average total operation time in the navigation group was lower than that in the non-navigation group (*P* < 0.01). All clinical outcomes showed improvement at different time points postoperatively, with no significant difference noted between the two groups (*P* > 0.05).

**Conclusions:**

Compared with the non-navigation approach, O-arm total navigation assistive BE-LIF technology not only has similar clinical results, but also can provide accurate intraoperative guidance and help spinal surgeons achieve accurate decompression. Furthermore, it can reduce radiation exposure to surgeons and operation time, which improve the efficiency and safety of surgery.

## Introduction

With the aging of the population, the number of patients with lumbar degenerative diseases (LDD) is gradually increasing. Minimally invasive spinal surgery (MISS) has been favored by spinal surgeons and patients in recent years because of less intraoperative trauma, less bleeding, less postoperative pain, and faster recovery (1). With the advancement of MISS, unilateral biportal endoscopic lumbar interbody fusion (BE-LIF) has become an alternative approach for treating LDD (2–4). Owing to separation of the endoscopic and instrument channels in the BE-LIF technique, the movement range of the endoscopic visual field is larger, and the instrument operation is more flexible. However, in the process of establishing the endoscopic and instrument channel, the instrument is sometimes not found due to the complex structure of spine anatomy (5). In addition, endoscopic surgery can allow a partially enlarged visual field under the endoscope; however, it cannot allow observing the surrounding anatomical reference objects under direct vision like open surgery, and the current anatomical location is unclear and easily lost in the field of vision, which has caused great confusion among doctors. Further, as with other minimally invasive endoscopic procedures, fluoroscopic assistance is essential for BE-LIF because it is required from the localization of the skin incision to the determination of the channel, satisfactory position of the fusion device in the intervertebral space, and placement of percutaneous pedicle screws (6, 7). Therefore, radiation exposure of surgeons is also a concern.

With the development of digital medical technology, the O-arm navigation system has been successfully applied in minimally invasive transforaminal interbody fusion (MIS-TLIF) and oblique lateral lumbar interbody fusion (OLIF), with good clinical results reported (8, 9). In addition, many studies have shown that they can effectively improve surgical efficiency and reduce radiation exposure in doctors (10, 11). However, to the best of our knowledge, the benefits of O-arm total navigation-assisted BE-LIF surgery have not been reported. Therefore, this study aimed to introduce BE-LIF assisted by intraoperative O-arm total navigation and compare it with traditional fluoroscopy-assisted BE-LIF to explore its strengths and weaknesses.

## Material and methods

### Study design and patient population

We retrospectively analyzed 44 patients with BE-LIF who were treated with O-arm total navigation assistance (21 patients) and traditional C-arm fluoroscopy assistance (23 patients) at our spine center from August 2020 to June 2021. This retrospective study was approved by the Ethics Committee of the Second Affiliated Hospital of the Army Medical University, and informed consent was obtained from all patients.

The inclusion criteria were as follows: (1) age ≥ 18 and ≤75 years, (2) definite diagnosis of single-level lumbar central canal stenosis with spondylolisthesis or instability and lumbar nerve root canal stenosis with spondylolisthesis or instability, (3) no response to appropriate conservative treatment over 3 months. The exclusion criteria were as follows: (1) anesthesia was not possible because of poor physical or mental condition; (2) high-grade (>grade 2) spondylolisthesis, spondylodiscitis, active infection, spinal fractures, history of lumbar surgery, and spine tumor. (3) Patients with less than 1 year follow-up and incomplete clinical data.

The choice of O arm or C arm-assisted BE-LIF was based on patients fully understanding the details, advantages and disadvantages, and total cost of the two methods, and making the final choice after fully considering communication with doctors and their own health insurance status. All the patients completed at least 12 months of follow-up. Perioperative data, including operative time, estimated intraoperative blood loss, postoperative drainage, postoperative hospital stay, radiation dose, and duration of radiation exposure, were collected for the navigation and non-navigation groups. Clinical outcomes were assessed using the visual analog scale (VAS) score (back pain score) and the Oswestry Disability Index (ODI) at baseline, 3 day, 3 months, 12 months after surgery. At the final follow-up, patient satisfaction was assessed according to the modified MacNab criteria (excellent, good, medium, or poor). Perioperative complications, including severe nerve root and epidural injuries, epidural hematoma, vascular injury, and incomplete decompression, were assessed. Spine fusion was evaluated by a radiologist using computed tomography (CT) images obtained at least 1 year after surgery. In the CT images, evident fusion was considered as bridging trabecular bone formation between the vertebral bodies.

### BE-LIF assisted by O-arm total navigation surgical procedure

**Figure 1:**
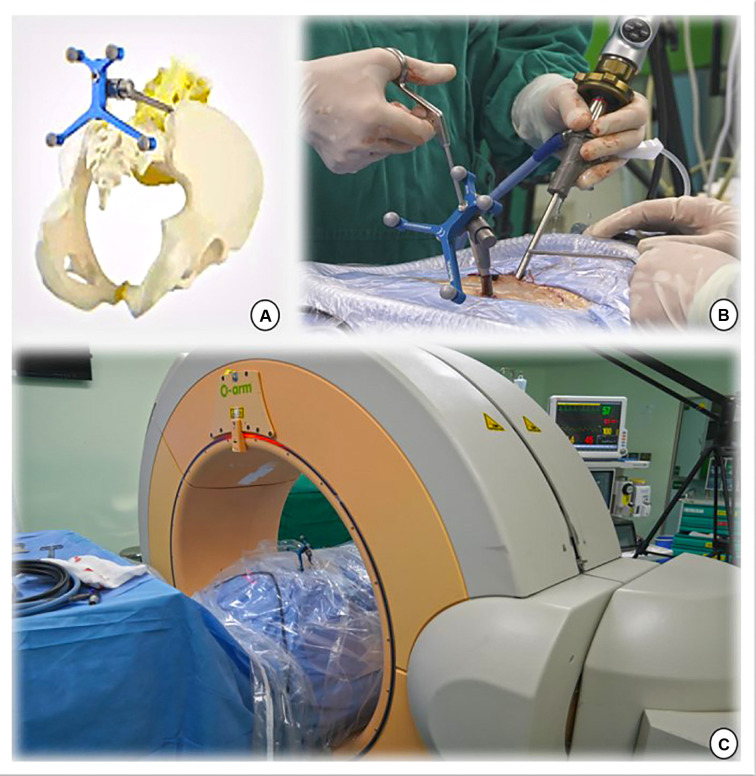
(**A,B**) The navigation reference frame is fixed to the posterior superior iliac spine. (**C**) The O-arm device is placed and prepared for image capture.

Under general anesthesia, the patient was placed prone on a radiolucent table. The reference frame was anchored to the iliac crest using a positioning needle (Figures 1A,B). Subsequently, an intraoperative CT scan and 3-dimensional (3D) images were obtained using the O-arm (O-arm Surgical Imaging System and Stealth-Station; Medtronic, Minneapolis, MN, USA) (Figure 1C). The obtained imaging data were automatically instantaneously transmitted to the navigation system, and a multiplanar image of the lumbar spine was reconstructed, including x-ray-like anteroposterior and lateral views, axial and sagittal planes of lumbar vertebrae, and even 3D images of the lumbar spine. Finally, the surgical instruments were registered to perform real-time tracking intraoperatively. Typically, the entire preparation process of the navigation system includes fixing the reference frame, O-arm scanning, image transmission, and tool registration in less than 10 min.

**Figure 2:**
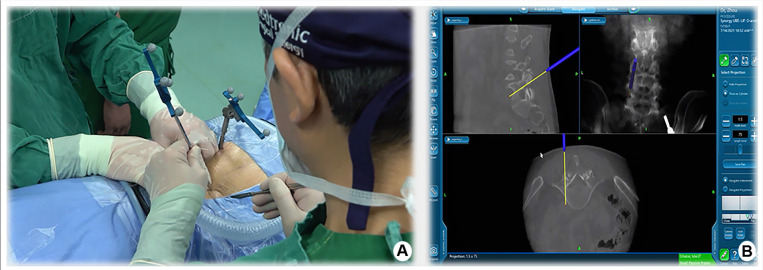
Design of intraoperative skin incision assisted by navigation.

**Figure 3:**
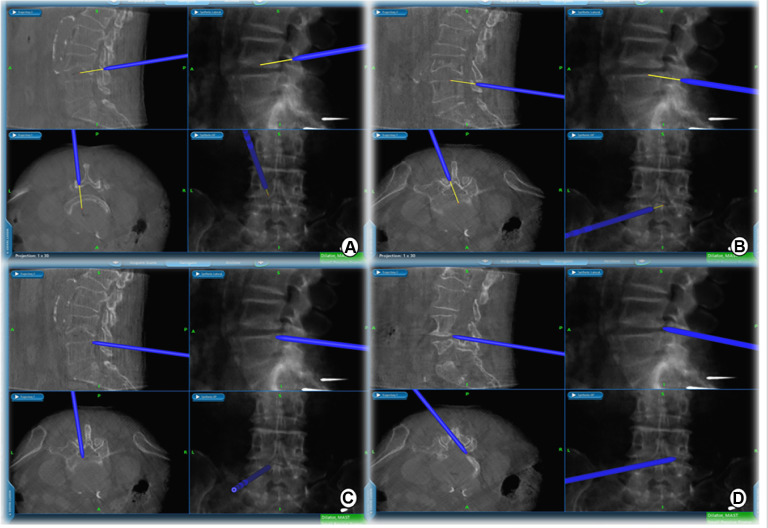
Extent of intraoperative decompression can be confirmed in real time on the navigation screen. (**A**) cranial, (**B**) caudal, (**C**) ipsilateral, and (**D**) contralateral decompression range detection probes.

**Figure 4:**
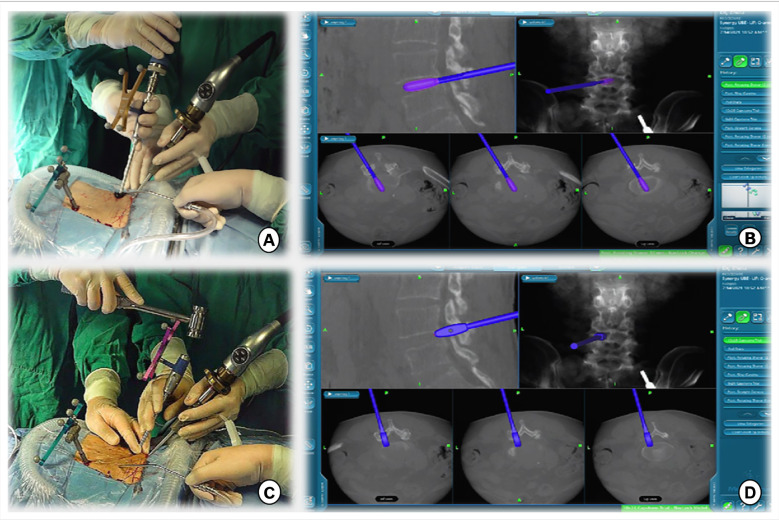
(**A,B**) Intervertebral space is processed with reamers of different diameters, whose angles and orientations can be displayed in real time on a computer screen. (**C,D**) A serial trial under navigation guidance was used to determine the height of the disc and true size of the CAGE.

**Figure 5:**
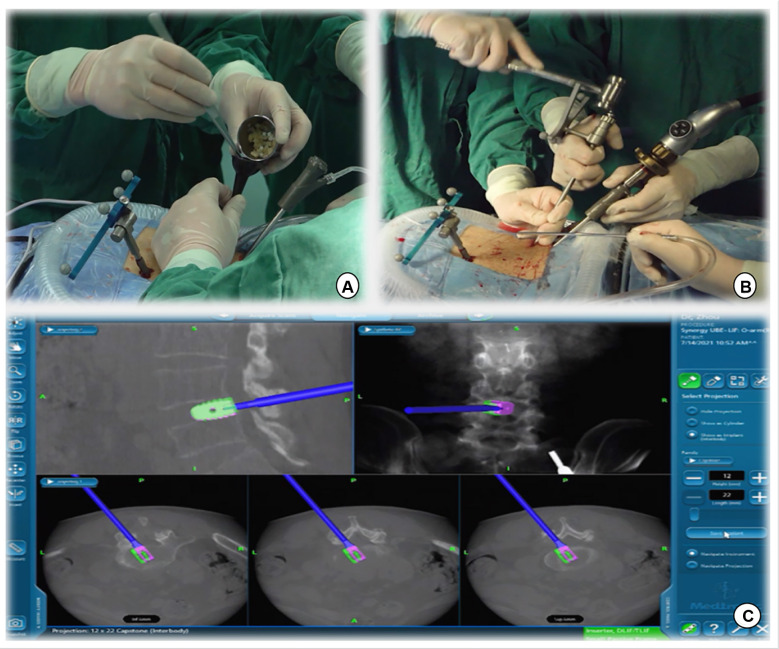
(**A**) Intervertebral bone grafting was performed using a specialized funnel. (**B,C**) The PEEK CAGE can be safely inserted into the intervertebral space with the size, orientation, and depth displayed on the navigation screen.

**Figure 6:**
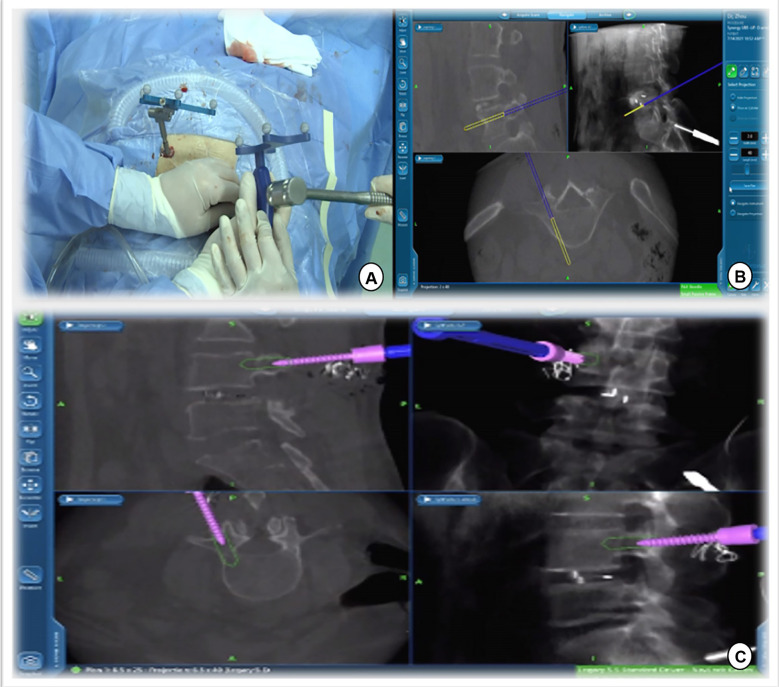
Track of the access tracker was visible in real time, and spine surgeons could adjust the needle entry point based on the trajectory and position image of the screw displayed in real time on the navigation screen.

Taking the left surgical approach as an example, two outward skin incisions were made at the medial edge of the ipsilateral upper and lower pedicle. The skin incision design can be displayed on the screen in real-time using a navigation probe, allowing doctors to adjust the incision position based on the image (Figure 2). After the incision of the skin and lumbar dorsal muscle fascia, sequential dilation through the two incisions was performed to separate the soft tissue covered by the lamina and facet surfaces. An arthroscopic system was inserted into the observation channel, and continuous lavage fluid was maintained from the endoscopic entrance to the working channel, followed by radiofrequency cleaning of the lamina surface soft tissue and control of bleeding through the working channel. Laminectomy and facet resection were performed under navigation using a high-speed drill, Kerrison punch, and ring saw. When decompression is required on the contralateral side, contralateral lamina and facetectomy are performed similarly. The extent of bone decompression was confirmed in real-time on the navigation screen (Figure 3). The resected lamina and facet joints were collected as autologous bone. The ipsilateral and contralateral ligamentum flavum were then removed to decompress the central canal and bilateral nerve roots, exposing the Kambin's triangle. Under navigation guidance, the intervertebral disc tissue was removed with pituitary forceps and reamers of different diameters, and the reamer angle and direction can be displayed on the computer screen in real-time (Figures 4A,B). The cartilage endplate was then removed using a curette under endoscopic visualization. Then, a serial trial under navigation guidance was used to determine the disc height and true size of cage (Figures 4C,D). The intervertebral disc was then filled with the harvested autologous local bone from laminectomy and facetectomy, recombinant human bone morphogenetic protein, and allografts through a specialized funnel cannula (Figure 5A). With the aid of navigation, the polyetheretherketone (PEEK) cage was placed at an appropriate depth in the intervertebral space (Figures 5B,C). The final positions of the PEEK cages were identified using the C-arm. Subsequently, O-arm scans were repeated. Percutaneous pedicle screw fixation was performed using two previous ipsilateral incision and two new contralateral incisions under navigation. The entry point of the pedicle screw can be adjusted according to the real-time image of the screw trajectory and the position displayed on the monitor (Figure 6). Finally, the C-arm is usually used to confirm the final position of the screw and to place the drain before the skin is sutured.

### Surgical technique of BE-LIF assisted by C-arm

This procedure was performed as a routine BE-LIF procedure, as reported previously (12). The radiation dose and duration of radiation exposure were collected from the radiation emitters immediately after surgery.

### Statistical analysis

The IBM SPSS Statistics Ver. 26.0 (IBM Co., Armonk, NY, USA) was used for statistical analysis. Data are presented as mean ± standard deviation or frequency. Clinical outcomes (VAS score and ODI for back and leg pain) between the two groups and changes over time in each group were analyzed using repeated-measures analysis of variance. The least significant difference (LSD) test was used to clarify the changes at different time points in the same group. Differences between the two groups were examined using the independent sample t-test, chi-square test, and Mann‒Whitney U test, as appropriate, based on different categories of data. Statistical significance was set at *P*-value <0.05.

## Results

No significant differences were noted in the demographic data between the navigation and non-navigation groups (Table 1). The radiation dose in the navigation group was 3.18 ± 1.02 mGy, which was significantly lower than that in the non-navigation group (14.38 ± 3.26 mGy) (*P* < 0.01). The radiation exposure time was 6.90 ± 2.30 s in the navigation group and 31.55 ± 5.88 s in the non-navigation group. No significant differences were noted in the estimated intraoperative blood loss, postoperative drainage, postoperative hospital stay, or perioperative complications between the two groups. The average total operation time (154.04 ± 11.17 min) in the navigation group was reduced compared with that in the non-navigation group (operation time, 170.91 ± 12.01, *P* < 0.001). There were no significant differences in the VAS score and ODI between the two groups at baseline. Compared with preoperative scores, the VAS score and ODI in both the groups improved significantly at different time points after surgery (Table 2). However, no significant differences were noted between the two groups. There was no significant difference in the excellent and good rates between the navigation (95.23%) and non-navigation groups (91.30%). We observed one dural tear and one transient ipsilateral dysesthesia in the non-navigation group, which recovered with conservative treatment. No major complications occurred in either group. Twelve months after surgery, the rate of spinal fusion was 82.60% (19 patients) in the non-navigation and 85.71% (18 patients) in the navigation group, with no significant difference between the two groups. We did not observe any case of spinal non-fusion at the last follow-up among any patient.

**Table 1:** Demographic and perioperative data.

Characteristic	Navigation (*n* = 21)	Nonnavigation (*n* = 23)	*P* value
Age (years)	57.71 ± 8.78	60.39 ± 9.14	0.329
Sex			0.599
Male	7	6	
Female	14	17	
Body mass index (kg/m^2^)	25.48 ± 1.66	26.09 ± 1.72	0.243
Diagnosis			0.744
Lumbar central canal stenosis with spondylolisthesis or instability	14	17	
Lumbar nerve root canal stenosis with spondylolisthesis or instability	7	6	
Surgical levels			0.770
L3–4	1	3	
L4–5	16	17	
L5–S1	4	3	
Operation time (mins)	154.04 ± 11.17	170.91 ± 12.01	<0.001
Radiation exposure duration (sec)	6.90 ± 2.30	31.55 ± 5.88	<0.001
Radiation dose (mGy)	3.18 ± 1.02	14.38 ± 3.26	<0.001
postoperative hospital stay	4.47 ± 1.03	4.95 ± 0.87	0.103
Intraoperative Estimated blood loss (ml)	93.23 ± 15.88	95.34 ± 16.28	0.666
Postoperative drainage (ml)	69.52 ± 19.61	77.82 ± 19.05	0.162
Complications (*n*)	0	2	0.489

Values are presented as number or mean ± standard deviation.

**Table 2:** Comparison of clinical outcomes between the two groups.

Clinical Data	Navigation	Nonnavigation	*P*-value
VAS scores of low back			0.690
Preoperative	6.09 ± 1.33	5.91 ± 1.41	0.663
Postoperative 3 Day	2.71 ± 0.78	3.04 ± 0.87	0.198
3 Months	1.52 ± 0.51	1.60 ± 0.58	0.612
12 Months	0.71 ± 0.63	0.95 ± 0.70	0.242
VAS scores of leg			0.215
Preoperative	5.47 ± 2.20	6.13 ± 2.02	0.311
Postoperative 3 Day	1.71 ± 1.61	2.04 ± 1.77	0.524
3 Months	1.38 ± 1.49	1.17 ± 0.93	0.582
12 Months	0.76 ± 0.88	0.82 ± 0.65	0.785
ODI scores			0.099
Preoperative	55.04 ± 9.35	52.26 ± 7.86	0.290
Postoperative 3 Day	21.52 ± 7.50	23.30 ± 9.05	0.484
3 Months	10.57 ± 3.58	12.17 ± 4.78	0.219
12 Months	7.80 ± 3.62	6.69 ± 2.60	0.245
Fusion rate (%)	85.7%	82.6%	1.000
MacNab criteria (*n*)			0.418
Excellent	16	13	
Good	4	8	
Fair	1	2	
Poor	0	0	

VAS, visual analogue scale; ODI, Oswestry Disability Index.

## Discussion

As a burgeoning minimally invasive endoscopic spine surgery, BE-LIF has been successfully applied to degenerative diseases of the lumbar spine with good clinical results (4, 13, 14). Compared with traditional open lumbar fusion surgery, BE-LIF involves less trauma, less postoperative pain, and faster recovery and is equally effective in improving clinical outcomes and achieving fusion (3, 15). However, as with other minimally invasive endoscopic procedures, BE-LIF only provides a locally magnified and clear field of view under endoscopy, and it is impossible to see the anatomical markers outside the field of endoscopy, which may cause the surgeon to be lost the field of view during the endoscopic operation, thus affecting the surgery efficiency. Furthermore, some studies have reported that incomplete decompression during surgery is an important factor leading to the failure of Unilateral biportal endoscopy (UBE) (16–18). In addition, percutaneous endoscopic fusion surgery leads to radiation exposure among doctors, which is always concerning. Therefore, improving the surgical efficiency of BE-LIF and reducing radiation exposure are significant problems.

Recently, O-arm navigation systems have been used in MIS-TLIF and OLIF (8, 19, 20). Based on imaging data, the O-arm assisted navigation system uses real-time technology to show the relationship between surgical instruments and the anatomy of the surgical area and assists surgeons in surgical operations (21, 22). In addition, navigation technology can improve the safety and accuracy of percutaneous screw placement and reduce the risk of intraoperative nerve roots, blood vessels, and various clinical complications (23, 24). However, endoscopic decompression and fusion are equally important in endoscopic spinal fusion surgeries. In our study, we demonstrated total navigation technology, which navigated the entire process from the location of the skin incision to endoscopic decompression and fusion and then to the placement of a percutaneous pedicle screw.

First, the surgical incision in the BE-LIF technique should consider the extent of decompression, placement of pedicle screws, and exposure of the intervertebral space to ensure the placement of CAGE (4). Appropriate incision positioning can make the surgical process more efficient, prevent additional incisions for percutaneous pedicle screw fixation, and minimize surgical trauma. Accurate localization of skin incisions requires repeated fluoroscopy, which increases radiation exposure and operation time. In this study, we implemented the application of navigation probes to facilitate body surface localization and accurate incision selection (Figure 2). It is well known that UBE technology is most commonly used to treat patients with lumbar spinal stenosis by unilateral laminectomy and bilateral decompression (25). Nevertheless, inadequate decompression was significantly associated with patient dissatisfaction in a multicenter cohort study of UBE surgical failure (26). Under the guidance of our total navigation system for intraoperative decompression, the orthopedic surgeon can observe the actual position of the instrument through three-dimensional images displayed on the navigation screen and the farthest safe position that the surgical tool can reach in real time, avoid unnecessary laminal and facetectomy, and achieve accurate decompression (Figure 3). After nerve decompression and intervertebral space exposure, adequate disc management and endplate preparation are key factors for endoscopic fusion. Theoretically, BE-LIF can be used to perform discectomy and endplate preparation using conventional spinal surgery tools under endoscopic monitoring (27, 28). However, in the actual operation, because the endoscope can only provide two-dimensional images, in the process of intervertebral space processing and CAGE trial and placement, only the tail end of the instrument can be seen sometimes, and it is impossible to judge whether its angle and direction are parallel to the endplate, which may cause damage to the end plate and affect the bone graft bed. Under navigation, the three-dimensional image of the instrument can be displayed in real-time. Figure 4 shows the use of a 12-mm reamer to manage the intervertebral space, which can clarify the angle and direction of the intervertebral space management process and observe the intervertebral space management range. The CAGE series of trials were performed after the cartilage endplates were managed with different tools. The direction and depth of entry were displayed on the navigation screen (Figures 4C,D), and the appropriate size was selected. The PEEK CAGE can be safely inserted into the intervertebral space, with the size, orientation, and depth displayed on the navigation screen, thereby avoiding endplate injury and misplacement (Figures 5B,C). Many studies have reported percutaneous pedicle screw process injury to the spinal cord and nerve roots (23, 29, 30). Surgeons often repeatedly adjust the trajectory direction of screws under fluoroscopy. Under navigation, spine surgeons could adjust the needle entry point based on the trajectory and position image of the screw displayed in real-time on the navigation screen (Figure 6), thereby avoiding repeated fluoroscopy.

BE-LIF assisted by O-arm navigation offers several benefits. First, in our study, there were no statistically significant differences in perioperative data except for radiation exposure and operative time. Clinical evaluation including VAS score, ODI, Modified MacNab criteria and Fusion rate were not significantly different in two groups. These results indicate that, compared with traditional C-arm assisted BE-LIF, O-arm navigation-assisted BE-LIF not only has similar clinical effects, but also can achieve accurate skin incision design, accurate intraoperative decompression, percutaneous pedicle screw placement, while reducing radiation exposure, which improves surgical efficiency. In addition, during endplate preparation and CAGE implantation, doctors can observe the angle and direction of instrument management, evaluate the range of intervertebral space management, and define the direction and depth of CAGE implantation, which improves surgery safety. No major complications occurred in either group. In the non-navigational group, one case of dural tear and one case of transient ipsilateral paresthesia were recorded, both of which had clinical symptoms that disappeared after conservative treatment. However, the potential clinical application risks include mechanical image drift caused by unstable reference frames and spinal structural errors caused by intraoperative traction soft tissue displacement, which may cause inaccurate navigation (21). Therefore, we often used C-arm fluoroscopy to confirm the accuracy of the navigation of key steps during the procedure. In addition, considering the possible changes in vertebral body shape after CAGE implantation, we performed a second scan before percutaneous pedicle screw placement, which consumed some time and increased radiation exposure. O-arm navigation does not prevent radiation exposure to the patient because it must remain in the radiation field during image acquisition. The higher doses exposed to patients and the prevention of intraoperative navigation image distortion are the concerns of this technology.

This study has several limitations. First, this is a single-center retrospective study, which may have led to a selection bias. Second, the number of cases was small, and the follow-up time was short. Prospective, multicenter, large-sample prospective studies are needed in the future.

## Conclusion

Compared with the non-navigation approach, O-arm total navigation assistive BE-LIF technology not only has similar clinical results, but also can provide accurate intraoperative guidance and help spinal surgeons achieve accurate decompression. Furthermore, it can reduce radiation exposure to surgeons and operation time, which improve the efficiency and safety of surgery.

## Data Availability

The raw data supporting the conclusions of this article will be made available by the authors, without undue reservation.
